# Systematics for types and effects of RNA variations

**DOI:** 10.1080/15476286.2020.1817266

**Published:** 2020-09-20

**Authors:** Mauno Vihinen

**Affiliations:** Department of Experimental Medical Science, Lund University, Lund, Sweden

**Keywords:** RNA variation classification, variation ontology, VariO, RNA, systematics

## Abstract

Systematics is described for annotation of variations in RNA molecules. The conceptual framework is part of Variation Ontology (VariO) and facilitates depiction of types of variations, their functional and structural effects and other consequences in any RNA molecule in any organism. There are more than 150 RNA related VariO terms in seven levels, which can be further combined to generate even more complicated and detailed annotations. The terms are described together with examples, usually for variations and effects in human and in diseases. RNA variation type has two subcategories: variation classification and origin with subterms. Altogether six terms are available for function description. Several terms are available for affected RNA properties. The ontology contains also terms for structural description for affected RNA type, post-transcriptional RNA modifications, secondary and tertiary structure effects and RNA sugar variations. Together with the DNA and protein concepts and annotations, RNA terms allow comprehensive description of variations of genetic and non-genetic origin at all possible levels. The VariO annotations are readable both for humans and computer programs for advanced data integration and mining.

## Introduction

RNA variations are increasingly in the focus of molecular biology, genetics and medicine. Known disease-causing RNA variants mainly affect proteins. Non-coding RNAs have gained interest and have been shown to have numerous functions. Nowadays, tens of types of RNA molecules are known. Those are functional or related to numerous cellular processes and functions ranging from catalysis to regulatory processes, from information transfer for protein synthesis to function in cellular machineries, from RNA base and sugar modification to RNA interactions, and so on.

Terminology for RNA molecules, functions and processes is still confusing. There is not even consensus for the definition of RNA – apart from being chemically a polynucleotide [1]. Common language and concepts are needed for efficient communication. In addition to human information transmission, computational analyses are not possible without systematic data presentation. Variation Ontology (VariO, http://variationontology.org/) was developed for systematic description of variations and their consequences, effects and mechanisms [2]. VariO includes terms for all kinds of alterations in DNA, RNA and protein. DNA and protein descriptions and systematics have been more mature than those for RNA, largely because novel types of RNA molecules, their functions and effects are reported continuously. Here, RNA terms and the hierarchical structure in VariO are discussed.

VariO annotations can be made on three molecular levels – DNA, RNA and protein – and for four types of information: variation type, structure, function, and properties. This article concentrates on RNA variations, detailed descriptions of DNA and protein variants have been published previously [3,4]. VariO annotations are made in relation to a reference state, which can be a reference sequence, wild-type property or similar. A new VariO version includes all the additions and modifications made during the preparation of this article. By combining VariO with other systematics even more detailed and nuanced systematic descriptions can be made.

When using VariO, systematic annotations, include the VariO prefix and a number followed by the term. The notation VariO:0319 means the same as the name of the term, i.e. ‘RNA deletion’. When using annotations, both the number and the prefix ‘VariO’ must be included. Although the names of the terms can be obtained automatically, full names must be provided when the information is intended to be read by people. This article follows the VariO RNA term hierarchy on the four sublevels (variation type, function, structure and properties). The headings and subheadings are VariO terms. For clarity, VariO terms are written in quotation marks in the text. We have published guidelines for the annotation process [5]. The variation type annotations can be generated automatically with VariOtator annotation tool [6] that makes it easier to obtain systematic and coherent annotations. The other types of annotations are made manually, the interactive VariOtator tool can be used for that purpose.

VariO is available in several ways in addition to the ontology website (http://variationontology.org/) including Ontology Lookup Service (https://www.ebi.ac.uk/ols/ontologies/vario), OBO Foundry (http://www.obofoundry.org/ontology/vario.html), NCBO BioPortal (https://bioportal.bioontology.org/ontologies/VARIO) Ontobee (http://www.ontobee.org/ontology/VariO), FAIRsharing (https://fairsharing.org/bsg-s000776/) and others.

Despite extensive databases for experimental RNA data are available, many features and properties of RNA molecules have to be addressed with computational prediction methods. Here, many of these tools are introduced in the context of the VariO annotations. The prediction method choice is an important step. Systematic benchmark performance assessments provide the most reliable information for the choice. The problem is that such studies are missing for many RNA related predictions, largely due to limited amount of known experimentally verified cases. Guidelines have been published for systematic reporting of predictor performance and implementation [7,8].

This article introduces the hierarchy and use of RNA related terms in VariO. The description of almost all the terms is followed by cases from literature or from databases to provide examples for the use of annotations.

## Databases for RNA variations

Several databases distribute RNA information, examples are shown in Table 1. Sequences in databases like GenBank and EMBL for coding sequences are for sense strand and thus identical to mRNA sequence except for containing thymine instead of uracil. Databases managed at Leiden Open Variation Database (LOVD) management system [9] are the major sources for genetic variation information. Strangely enough, RNA level descriptions are missing from the majority of LOVD databases, even when protein changes are included. IDbases for primary immunodeficiencies [10] are an exception as they include also the RNA changes.

**Table 1.** Databases for RNA information

Database	URL	Reference
RNA variation databases		
RNAcentral	https://rnacentral.org	[13]
Alternative polyadenylation site database APASdb	http://genome.bucm.edu.cn/utr/	[14]
Splicing databases		
ASpedia	http://combio.snu.ac.kr/aspedia	[15]
DBASS3 and DBASS5	http://www.dbass.org.uk/	[16]
ExonSkipDB	https://ccsm.uth.edu/ExonSkipDB/	[17]
MiasDB	http://47.88.84.236/Miasdb/	[19]
SASD	http://bioinfo.hsc.unt.edu/sasd	[18]
Short non-coding RNAs		
DASHR	http://dashr2.lisanwanglab.org/	[20]
miRmine	http://guanlab.ccmb.med.umich.edu/mirmine	[21]
miRpathDB	https://mpd.bioinf.uni-sb.de/	[23]
miRTarBase	http://miRTarBase.cuhk.edu.cn/	[22]
miRandb	http://miRandb.ir	[26]
HMDD	http://www.cuilab.cn/hmdd	[24]
miRwayDB	http://www.mirway.iitkgp.ac.in	[25]
piRBase	http://www.regulatoryrna.org/database/piRNA/	[27]
piRDisease	http://piwirna2disease.org/	[28]
Long non-coding RNAs		
LncVar	http://bioinfo.ibp.ac.cn/LncVar	[29]
LnCeVar	http://www.bio-bigdata.net/LnCeVar/	[11]
LncCeRBase	http://lnccerbase.it1004.com	[30]
LncRNADisease	http://www.rnanut.net/lncrnadisease/	[34]
LIVE	https://live.bioinfotech.org References	[32]
lncRNASNP	http://bioinfo.life.hust.edu.cn/lncRNASNP2	[33]
tRNA		
T-psi-C	http://tpsic.igcz.poznan.pl	[35]
Circular RNA		
circRNA disease	http://cgga.org.cn:9091/circRNADisease/	[37]
Circ2Traits	http://gyanxet-beta.com/circdb/	[36]
RNA modifications		
MODOMICS	http://modomics.genesilico.pl	[38]
Editome Disease Knowledgebase (EDK)	http://bigd.big.ac.cn/edk	[40]
LNCediting	http://bioinfo.life.hust.edu.cn/LNCediting/	[42]
siRNAmod	http://crdd.osdd.net/servers/sirnamod	[39]
REDIportal	http://srv00.recas.ba.infn.it/atlas/	[41]
MeT-DB	http://compgenomics.utsa.edu/MeTDB/	[43]
RNA localization		
RNALocate	http://www.rna-society.org/rnalocate/	[44]
RNA structure		
Rfam	http://rfam.org	[45]
RNAStructuromeDB	https://structurome.bb.iastate.edu	[46]
RNApdbee	http://rnapdbee.cs.put.poznan.pl/	[47]
RNArchitecture	http://iimcb.genesilico.pl/RNArchitecture/	[49]
ChiTaRS	http://chitars.md.biu.ac.il/	[Balamura^54^]
BGSU RNA Site	http://rna.bgsu.edu/rna3dhub/	
Nucleic Acid Database	http://ndbserver.rutgers.edu/	[48]
Cancer variation databases		
ChiTaRS	http://chitars.md.biu.ac.il/	[Balamura^54^]
ym500v3	http://ngs.ym.edu.tw/ym500/	[50]
Lnc2Cancer	http://www.bio-bigdata.net/lnc2cancer	[52]
ncRNA-eQTL	http://ibi.hzau.edu.cn/ncRNA-eQTL	[51]
CRlncRNA	http://crlnc.xtbg.ac.cn	[53]
SomamiR DB	http://compbio.uthsc.edu/SomamiR/	[12]

RNAcentral [13] is a portal that contains various information for non-coding RNAs originating from 28 databases. APASdb [14] is a dedicated resource for alternative polyadenylation sites. Many RNA molecules are matured by splicing, there are many databases about different aspects of splicing and splicing products. ASpedia [15] contains data for alternative splicing in human and DBASS3 and DBASS5 [16] for disease-related alternatively spliced RNAs. ExonSkipDB [17] is a registry for one special form of alternative splicing. SASD [18] was developed for proteomics studies to detect consequences of alternative splicing. In MiasDB there are data for interactions of molecules involved in alternative splicing [19].

Numerous web services are available for short and long non-coding RNAs (ncRNAs). In DASHR [20] there are genes and sequences as well as tissue and cell type information for six types of ncRNA sequences. Several databases have been developed for microRNAs (miRNAs) including miRmine for expression profiles [21], miRTarBase [22] for target details, miRPathDB [23] for target and pathway details, HMDD [24] for disease associations and miRwayDB [25] for miRNA pathway associations in diseases. miRandb combines various data items for miRNAs [26]. piRBase [27] contains sequence and function annotations and piRDisease disease associations for piwi interacting RNAs (piRNAs) [28].

LncVar contains variations in long non-coding RNAs (lncRNAs) [29]. lncRNAs are called for competing endogeneous RNAs (ceRNAs) when they bind to miRNA and regulate their functions. In LncCeRBase [30] there are details for ceRNAs, and lncRNAs containing genomic variants that disturb ceRNA network regulation [31]. All kinds of interactions of lncRNAs are available in LIVE [32]. Variations in lncRNAs can be found from lncRNASNP [33] and disease-associations from LncRNADisease [34].

T-psi-C is a database for tRNA sequences and structures [35]. Information about circular RNA (circRNA) disease associations can be obtained from Circ2Traits [36] and circRNA disease [37] databases.

In addition to splicing, RNAs undergo various other modifications, data for modifications, pathways, modifying enzymes and modification locations within sequences are available in MODOMICS [38]. siRNAmod [39] contains modified siRNAs. RNA editing-related resources include EDK for disease associations [40], REDIportal for human A to I editing events [41], and LNCediting for functional effects of lncRNA editing [42]. Data for N6-methyl-adenosine methyltranscriptome is available in MeT-DB [43].

Subcellular localization of RNA molecules varies, details are in RNALocate [44]. RNAs have been grouped to families based on sequence analysis in Rfam [45]. Rfam contains some secondary structure data, predicted secondary structural information can be found also from RNAStructuromeDB [46]. Experimentally determined RNA structures along with other details are in RNApdbee [47]. Nucleic Acid Database (NDB) [48] is a similar central portal as Protein Structure Database (PDB) for experimentally determined protein structures. Many RNA structures are also in PDB.

RNA families and structures are classified in several levels in RNArchitecture [49]. BGSU RNA Site contains 3D structure information organized into RNA Structure Atlas, RNA 3D Motif Atlas and Representative Sets of structures.

Several resources have been developed for RNA data in relation to cancers. YM500v3 [50] contains many data items for short RNAs and RNA-Seq datasets and information for their relevance to cancer. Another ncRNA resource is ncRNA-eQTL for expression profiles [51]. Lnc2Cancer [52] and CrlncRNA [53] contain experimentally supported lncRNA-cancer associations. Chimeric RNA transcripts, their three-dimensional contact maps and relevance for druggability can be searched from ChiTarRS [Balamura^54^].

## RNA variation type

**Figure 1.**
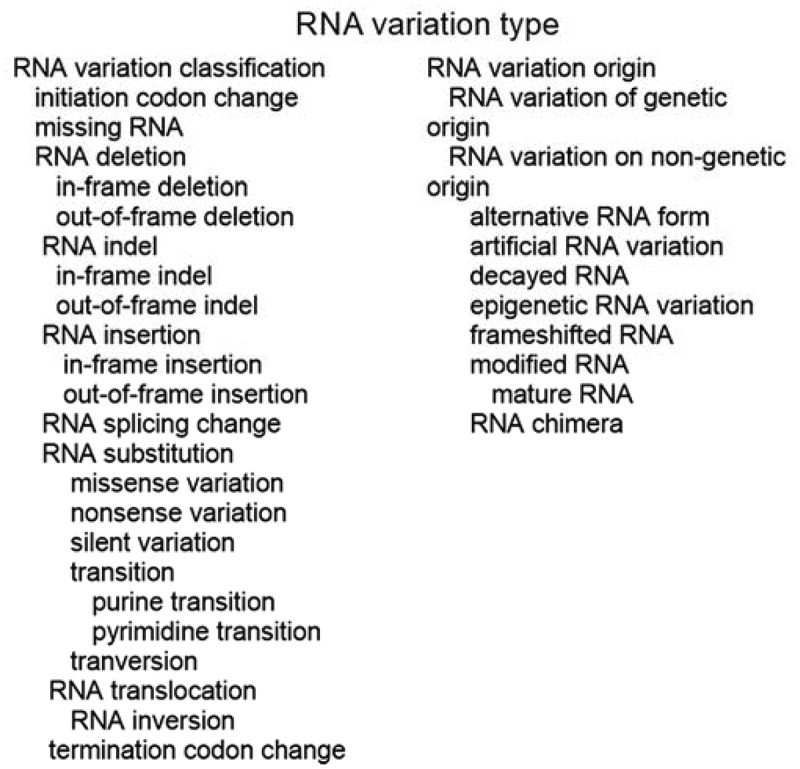
RNA variation types and division to RNA variation classification and variation origin terms. The hierarchy of the terms is indicated by indentation

The first category is ‘VariO:0306 RNA variation type’, which provides a brief description of the variation with commonly used terms (Fig. 1). These terms are not intended to replace Human Genome Variation Society (HGVS) names [55] or the International System for human Cytogenetic Nomenclature (ISCN) [56] instead to complement them. Naming conventions are an example of additional systematics used to provide rich and informative annotation. VariO terms provide simple, yet comprehensive descriptions. There are two sublevels for the RNA variation type descriptions: RNA variation classification and RNA variation origin (Fig. 1). There are examples for almost all the terms (a representative example may be shown for one term in a case of very similar terms). In the examples, HUGO Gene Nomenclature Committee (HGNC) names [57] were used for genes. Variants are indicated by prefix r. in RNA, when gene, coding region, mitochondrial or protein variants are discussed, the prefix is g., c., m. or p, respectively.

### VariO:0328 RNA variation classification

**Figure 2.**
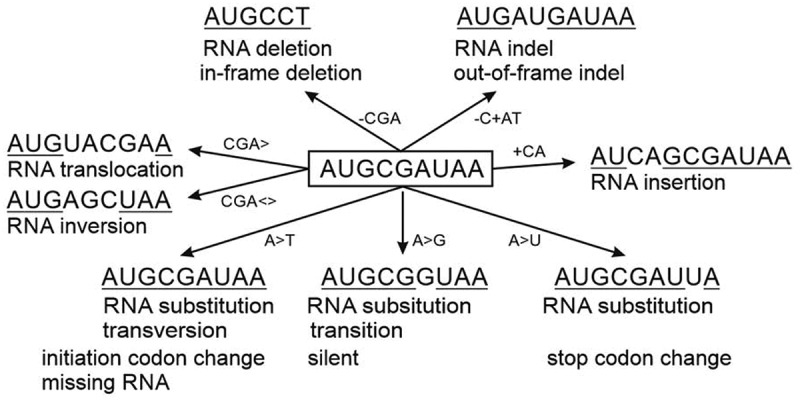
Examples of RNA chain variations. The original sequence is in the centre. In the variant sequences the original bases at original positions are underlined. In the coding region, deletions, indels and insertions are either in-frame or out-of-frame type. Nonsense variation that introduces a new, premature stop codon is not included. Similarly, RNA splicing change is omitted, see Fig. 5 for details

There are eight categories of RNA chain variation types, some of them with subcategories (Figs. 1 and 2). r.3g>u substitution in *BTK* [58] can be annotated as ‘VariO:0317 initiation codon change’, it is also ‘VariO:0312 RNA substitution’ of type ‘VariO:0316 transversion’. This variation prevents Bruton tyrosine kinase (BTK) expression and causes X-linked agammaglobulinemia (XLA). RNA substitution is either of transversion or of ‘VariO:0313 transition’ type. Transitions are further categorized as ‘VariO:VariO:0315 purine transition’ or ‘VariO:0314 pyrimidine transition’. RNA substitutions are further categorized based on the effect to coding region. ‘VariO:0308 missense variation’ is an RNA variation that causes amino acid substitution at protein level. Amino acid substitutions are frequently and erroneously called as missense variants [59]. The sense in the name refers to the information in mRNA triplet code. r.1559g>a missense variation due to c.1559G>A substitution causes p.R520Q substitution in BTK [60]. In ‘VariO:0310 nonsense variation’ the RNA substitution produces premature stop codon that leads either to truncated or completely missing protein. r.1135c>u transition due to c.1135C>T alteration leads to premature stop by introducing TAA codon in the middle of the *BTK* coding region [61]. ‘VariO:0318 silent variation’ does not change the coding region. r.954t>c alteratio rs5991926). Both the original and variant codon are for S318. Silent variations are synonymous due to the redundancy of the genetic code.

RNA quality control mechanisms, like nonsense-mediated decay (NMD) and staufen-mediated decay (SMD), degrade mRNA molecules that contain premature stop codons. Homozygous c.2978delG variation in *NPC1* coding for NPC intracellular cholesterol transporter 1 causes Nieman-Pick type C disease due to ‘VariO:0245 missing RNA’ [62]. NMD reduces the amount of mRNA in patients with *NPC1* variants with premature stops.

Substitution c.839+5G>A in *BTK* gene causes ‘VariO:0319 RNA deletion’ r.777_839del [63,64] and subsequent deletion of 21 residues from the protein leading to XLA. This deletion is of type ‘VariO:0320 in-frame deletion’. mRNAs containing ‘VariO:00321 out-of-frame deletion’ are more common as the length of the in-frame deletion has to be divisible by three to retain the reading frame and are thus rarer. Out-of-frame deletion-containing RNAs are typically destroyed by NMD unless the variant is located towards the end of the coding region. c.1953del in *BTK* gene leads to p.L652* protein truncation and XLA [65] due to ‘VariO:00321 out-of-frame deletion’. In-frame and out-of-frame terms are relevant only on the coding regions in mRNAs and not for variants in other types of RNAs and not for protein variants [59].

‘VariO:0311 RNA indel’ contains both inserted and deleted components. An example is r.1682_1683delinsa in *BTK* [66] which is also ‘VariO:0031 out-of-frame indel’. r.1401_1402delinsuu in *BTK* [67] is a ‘VariO:0030 in-frame indel’. This variation causes a ‘VariO:0029 sequence retaining amino acid indel’ in BTK protein and leads to XLA.

*BTK* r.1812_1813insgacagu is a ‘VariO:0326 RNA insertion’ introducing additional nucleotides. This XLA-causing variant [61] is of type ‘VariO:0332 in-frame insertion’. The other type of coding region insertions is ‘VariO:0327 out-of-frame insertion’.

When a sequence stretch in RNA is moved to a new location it is called for ‘VariO:0241 RNA translocation’. In ‘VariO:0244 RNA inversion’ sequence is inverted to its original place. If the stop codon is altered to code for an amino acid ‘VariO:0309 termination codon change’ occurs. r.1195u>c transition in *KISS1R* for KISS1 receptor 1 modifies stop codon and causes normosmic congenital hypogonadotropic hypogonadism [68].

### Vario:0324 RNA variation origin

RNA variation origin has two subclasses, ‘VariO:0307 RNA variation of genetic origin’ and ‘VariO:0333 RNA variation of non-genetic origin’ (Fig. 1). Variants of genetic origin appear at DNA level and are copied to RNA. *BTK* r.1559g>a missense variation leading to p.P520Q substitution [60] is of genetic origin.

Several variation types are of non-genetic origin. Adenosine deaminase (*ADA*) gene has nine alternative transcripts which are of type ‘VariO:0329 alternative RNA form’, see LRG_16 in Locus Reference Genomic database [69]. r.1442g>c substitution causing p.C481S substitution in a *BTK* construct is a ‘VariO:0247 artificial RNA variation’ [70]. c.2978delG variation in *NPC1* is recognized by NMD [62] making the RNA ‘VariO:0335 decayed RNA’. ‘VariO:0334 epigenetic RNA variation’ has been included for consistency, although such cases are not known. This is not to be confused with cases where RNA molecules are involved in silencing and regulation of DNA expression.

Many types of RNA molecules are heavily modified and alterations to modifications are related to many human diseases ranging from neurological diseases to diabetes, cancer and mitochondrial diseases [71]. A to I RNA editing modifies RNA sequence and is involved, e.g., in breast cancer [72]. This is a form of ‘VariO:0336 modified RNA’. ‘VariO:0436 mature RNA’ is an RNA form that has been completely modified. mRNA molecules used for translation are examples of mature RNAs.

During translation, ribosomes read mRNA molecules in triplets. When ribosome slips back one base pair or forward one base pair happens ribosomal frameshifting. As the consequence, the translated protein is different from this site onwards. In some organisms this process is intentionally used to generate more than one type of protein from a single mRNA with programmed frameshifting. It is beneficial especially for virus genomes, which must be compact to fit into the capsid. Random frameshifting leads to amphigoric protein sequence if the protein is translated. Human immunodeficiency virus 1 (HIV-1) gag-pol fusion protein [73] is due to ‘VariO:0409 frameshifted RNA’.

HIV-1 *gag-pol* is also an example of ‘VariO:0408 RNA chimera’. Chimeras can emerge with different mechanisms including, e.g., read-throughs of adjacent genes, juxtaposition of transcripts encoded by genes on different chromosomes, and from noncontiguous genes within the same chromosome. Human yippee like 5 (*YPEL5*) and protein phosphatase 1 catalytic subunit beta (*PPP1CBI*) genes form recurrent and reciprocal chimeras in chronic lymphocytic leukaemia [74].

## Variation affecting RNA function

‘VariO:0396 variation affecting RNA function’ has six categories. Mitochondrial m.1616A>G substitution in tRNA-Val gene and its RNA form r.1616a>g cause mitochondrial myopathy, encephalopathy, lactic acidosis and stroke-like episodes (MELAS) syndrome [75]. It is an example of ‘VariO:0401 effect on amino acid transfer of RNA’. Ribozymes are RNA molecules with catalytic activity. These molecules are involved in sequence-specific intramolecular cleavage of RNA. r.a28u transversion inactivates hammerhead ribozyme [76] and has a ‘VariO:0398 effect of catalytic RNA activity’. miRNAs silence genes and regulate gene expression. miR-140 regulates expression of several genes in chondrocytes. Seed region variation r.24a>g in *MIR140* leads to gain of function activity and causes human skeletal dysplasia [77] due to ‘VariO:0400 effect on regulatory function of RNA’.

r.3g>u substitution in *BTK* [58] initiation codon stops the flow of genetic information via RNA to protein and has a ‘VariO:0402 effect on RNA information transfer’. r.777_839del in-frame deletion in *BTK* causes XLA because of deletion of residues 260–280 from the protein distorts the structure of the Src homology 3 (SH3) domain [64]. This is an example of ‘VariO:0397 effect on RNA splicing function’ due to exon skipping. r.1559g>a missense variation causes p.R520Q substitution in BTK [60] as ‘VariO:0399 effect on translation’.

## Variation affecting RNA property

Variations affect several properties of RNAs and have general annotation ‘VariO:0298 variation affecting RNA property’. XLA-causing r.1559g>a missense variation in *BTK* has ‘VariO:0304 association of RNA variation to pathogenicity’ [60]. It has also ‘VariO:0302 conservation of variation site’ property as the position is highly conserved. Nieman-Pick disease-causing c.2978delG variation in *NPC1* mRNA is degraded by NMD and thus has ‘VariO:0010 effect on RNA abundance’ [62].

RNA catalytic activity changes are described with ‘VariO:0439 effect on RNA activity’ and terms at three more specific terms. Replacements at conserved adenosines 248 or 249 in the J5/15 region of RNase P, a ribozyme, have ‘VariO:0441 effect on RNA affinity’ [78]. Variants were introduced to *Tetrahymena thermophila* group I ribozyme to optimize and to have ‘VariO:0442 effect on RNA specificity’ [79]. These variants have also ‘VariO:0440 effect on RNA reaction kinetics’.

Degradation of RNA molecules is important for control of their function. Most RNA molecules are short-lived. m.1625c>t substitution in mitochondrial tRNA-Val gene *MT-TV* leads to very low steady-state levels (<1%) of normal mt-tRNA-Val because the variant tRNA remains deacylated and is rapidly degraded [80]. Individuals who carry the variant have profound metabolic disorder that often causes neonatal death due to ‘VariO:0299 effect on RNA degradation’. Repeats of pentapeptide microsatellites in the shared exon of brain expressed associated with NEDD4 1 (*BEAN1*) and thymidylate kinase 2 (*TK2*) are responsible for spinocerebellar ataxia type 31 (SCA31) [81]. These RNAs are toxic and form aggregates called RNA foci that disrupt structure of RNA-binding proteins and have ‘VariO:0300 effect of RNA folding’ at RNA level.

Iron-responsive elements (IREs) are RNA interaction motifs. Variations at IRE in the transcripts for ferritin light chain (*FTL*) gene cause hereditary hyperferritinemia – cataract syndrome (HHCS) with increased serum ferritin levels and early-onset cataracts [82]. IRE motifs in RNA interact with IRE binding proteins, which regulate the translation and stability of target transcripts in the iron metabolic pathway. r.g41c variant (Verona) in the IRE region causes ‘VariO:0305 effect on RNA interaction’.

CNG-triplet repeats (N indicating any nucleotide) are frequently behind neuromuscular diseases. CUG-repeats in DM1 protein kinase (*DMPK*) transcripts form labile aggregates and are annotated with ‘VariO:0364 effect on RNA aggregation’ [83].

Many mRNAs that are directed to compartments contain one or multiple localization signal sequences (zipcodes), which are recognized by zipcode binding proteins. Diaphanous-related formin 1 (*DIAPH*) mRNA localizes to endoplasmic reticulum in fibroblasts independent of zipcodes. Frameshift-causing variant in *DIAPH* loses perinuclear localization of the transcript [84], hence ‘VariO:0363 effect on RNA localization’. HHSC-causing *FTL* double variant r.18c>u, r.22u>g (Pavia2) has ‘VariO:0301 effect on RNA stability’ since it reduces the thermal stability of the IRE-containing RNA.

## Variation affecting RNA structure

RNA structure and architecture have several levels and there are large differences in the structures as there are different forms of RNA and of widely different sizes ranging from short polynucleotides, like siRNAs, to long noncoding RNAs and RNA genomes i.e. from less than 20 nucleotides to molecules of millions of nucleotides. In addition to the single-stranded form there are double and multiple stranded RNA forms.

r.22u>c transition at the D-stem in mitochondrial *MT-TL1* for tRNA-Leu(UUR) is related to hypertension because of ‘VariO:0308 variation affecting RNA structure’ [85].

### VariO:0349 affected RNA type

**Figure 3.**
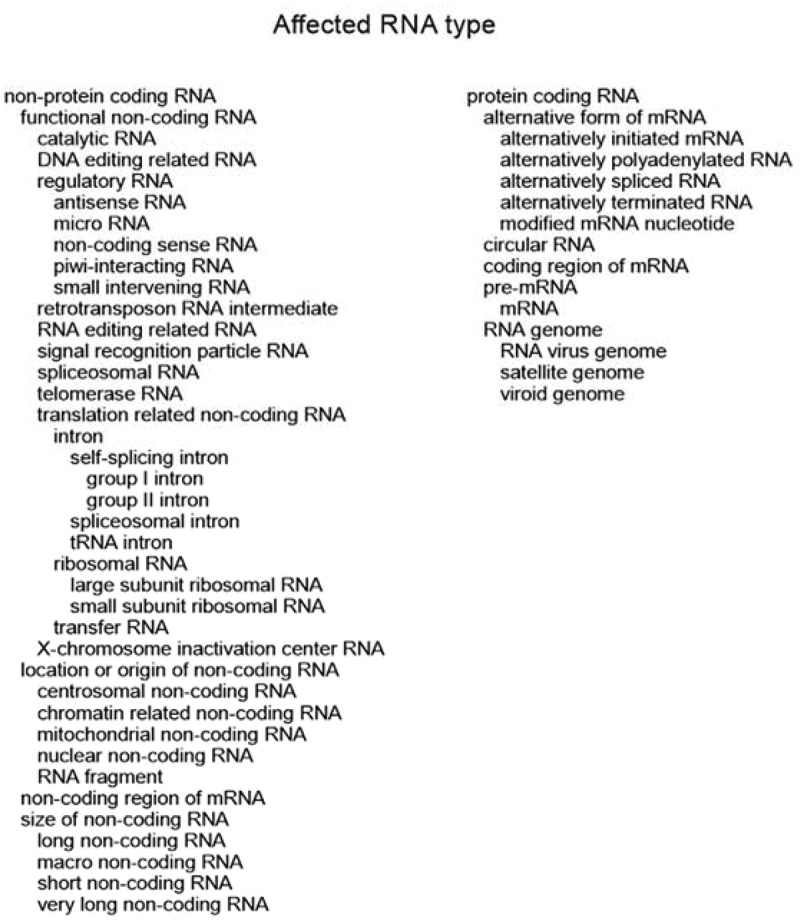
Affected RNA types of non-coding and protein-coding terms. The hierarchy of the terms is indicated by indentation

Genomes in many organisms are pervasively transcribed to large spectrum of RNA forms. There are two major types of affected RNA type, namely ‘VariO:0350 non-protein coding RNA’ and ‘VariO:0351 protein coding RNA’, see Fig. 3. HGNC provides official gene symbols. Now they are working also on non-coding RNAs, currently there are systematic names for more than 7000 RNA genes in 9 categories [86].

### Non-protein coding RNA

Classification principles have been presented for long non-coding RNAs [87,88], however, these schemes have included concepts and levels that are not readily comparable and thus a new systematic classification is introduced for all non-coding RNAs as well as for coding RNAs, see Fig. 3. There are six categories for non-coding RNAs including antisense and sense RNA, as well as untranslated region of protein-coding mRNA. Functional non-coding RNAs contain several groups, and classification based on location or origin of the RNA allows further details. The length of the RNA is an important factor, thus for their grouping there are terms based on the size of the polynucleotide. The goal of the classification is to include all types of ncRNAs, however, not to have terms to name all of them, because the field is rapidly developing and for annotation can be used other systematics, as well. Further, the terminology has not yet been established for all the transcripts.

Antisense RNA molecules are complementary to mRNA region, while sense RNA overlaps with mRNA, but is not involved in protein coding. Both are single-stranded molecules. ‘VariO:0463 Antisense RNA’ can block translation by hybridizing with mRNA. These transcripts are important regulators of protein expression and have biotechnological and therapeutic applications. The 3ʹ UTR region of DM1 protein kinase (*DMPK*) gene contains CTG repeats. Healthy people have few tens of repeats while patients with myotonic dystrophy type 1 (DM1) have more than 100 repeats, often even thousands of them. The repeats are part of CTCF insulator that regulates the expression of DMPK. An antisense transcript extends from the regulatory region of the adjacent gene to the CTF repeats. Extensive number of repeats affects the length of the antisense transcript and thereby gene regulation [89].

The regulation of *DMPK* expression is complex and involves also sense RNA, which is single-chain RNA that overlaps mRNA but is non-coding and annotated as ‘VariO:0464 sense RNA’ [89]. Congenital myotonic dystrophy (CDM) is the most severe form of the *DMPK*-related diseases. Analysis of a mouse model indicated that sense RNA, which contains the extended CTG repeats and surrounding regions, forms foci and co-localizes with muscleblind like splicing regulator 1 and 2 (MBNL1 and MBNL2) proteins [90]. The produced sense RNAs have unusual structures and aggregate together with the proteins and thereby affect numerous regulatory processes.

‘VariO:0353 non-coding region of mRNA’ in the mature mRNA contains the 3ʹ and 5ʹ flanking regions, which are essential, e.g., for regulation and translation.

#### Functional non-coding RNA

‘VariO:0465 Functional non-coding RNA’ has several subclasses (Fig. 3). Several computational tools have been developed for identifying functional RNAs, however not discussed in here because of being outside the topic, i.e., variations in the molecules. Ribozymes catalyse sequence-specific intramolecular cleavage. Variants like r.28a>u transversion in hammerhead ribozyme [76] appear in ‘VariO:0476 catalytic RNA’.

Genome editing, most notably with systems developed from the bacterial antiviral defence system with CRISPR/Cas9, is now widely used in research laboratories and biotechnological applications are under development. Editing is facilitated by guide RNA, a form of ‘VariO:0484 DNA editing-related RNA’ that directs the Cas9 nuclease to digest and remove or add new genetic material. The guide RNA detects a specific DNA location to be edited based on sequence complementarity. One of the early examples is the correction of a β-thalassaemia-causing double variant in human haemoglobin subunit beta (*HBB*) gene [91]. This variant is common among Chinese patients. The first variant is c.-78A>G in the promoter and the other c.126_129del deletion of 4 nucleotides coding for codons 41 and 42.

mRNA editing in humans and many other organisms is not RNA guided. Small nucleolar RNAs (snoRNAs) in many organisms and guide RNA (gRNA) in kinetoplastid protists are involved in RNA editing as ‘VariO:0483 RNA editing-related RNA’. snoRNAs guide methylation and pseudouridylation especially of rRNAs. Deletion of two bp (TT) from the small nucleolar RNA, C/D box 50A (*SNORD50A*) gene appears frequently in prostate [92] and breast cancer [93] cells. The variant is homozygous in prostate cancer but often heterozygous in breast cancer. *SNORD50A* expression is reduced due to the variation. The expression of wild-type form inhibits cancer cell growth.

Retrotransposons are genetic elements that can amplify themselves via ribonucleoprotein complex where the RNA transcript is reverse transcribed and integrated into a new position in the genome. The RNA transcript is a ‘VariO:0466 retrotransposon RNA intermediate’. Only a very small number of retrotransposons in a genome is transposable after insertion [94].

Several transcripts act as ‘VariO:0477 regulatory RNA’. miRNAs are typically about 22 nucleotides long RNA molecules that have a distinctive stem-loop structure. They regulate and silence gene expression. *MIR140* regulates expression of many chondrocyte genes. Seed region recognizes target mRNAs. Variation r.24a>g in *MIR140*, annotated as ‘VariO:0478 microRNA’, causes human skeletal dysplasia [77]. Several methods are available for miRNA target prediction and some for predictions of effects of variants in miRNA sequences [95,96]. Other line of tools addresses miRNA-disease associations [97–99]

‘VariO:0479 small intervening RNA’, abbreviated as siRNA, is 20–25 bp long double-stranded RNA. It functions in RNA interference (RNAi) pathway to regulate gene expression by directing targeted mRNAs for degradation and thus preventing protein production. Although siRNAs can be designed to silence disease-related variants, it is a daunting task where various aspects and predictions have to be taken into account [100]. This article reviewed also available computational tools. The performance of methods for bacterial small RNA target prediction has been benchmarked [101]. For siRNA silencing efficacy prediction there are many tools [102–105]

‘VariO:0480 Piwi-interacting RNA’, piRNA, interacts with piwi-subfamily Argonaute proteins that are mainly involved in post-transcriptional silencing of transcripts for repeat sequences, especially of transposable elements.

mRNA is spliced in a large protein-RNA complex that contains ‘VariO:0485 spliceosomal RNA’. Homozygous g.55G>A variation in *RNU4ATAC* gene for RNA, U4atac small nuclear (U12-dependent splicing) caused microcephalic osteodysplastic primordial dwarfism type I (MOPD I) with associated pigmentary disorder [106]. The encoded U4atac is a minor spliceosomal RNA. Signal recognition particles are evolutionarily conserved protein-RNA complexes in cytoplasm. They recognize and target specific proteins to plasma membrane in prokaryotes during translation and to endoplasmic reticulum in eukaryotes. They contain ‘VariO:0481 signal recognition particle RNA’.

*Xist* is a lncRNA and ‘VariO:0482 X-chromosome inactivation center RNA’ functional in X-chromosome inactivation (XCI) where one copy of the X-chromosomal genes is inactivated in females to compensate for the presence of only one copy in males. Deletion of one of the highly conserved Xist-specific repeat elements, repeat E, increases the expression of a number of XCI escape genes [107].

Telomeres are repetitive regions in the termini of chromosomes. They protect the chromosomes and are shortened gradually during chromosome replication as the Okazaki fragment binding region is not copied. Telomerase is a ribonucleoprotein complex that adds telomere repeats to the 3ʹ ends of telomeres to compensate for loss of sequence during replication. ‘VariO:0475 telomerase RNA’ is used as the template for repeats. Variations in telomerase RNA are associated for example to dyskeratosis congenita and aplastic anaemia. Cellular experiments show that disease-associated point variants in the pseudoknot and template regions of telomerase RNA lead to lower level of the RNA and much shorter telomeres [108].

Several RNA components act as ‘VariO:0468 translation-related non-coding RNA’. Proteins are synthesized at ribosomes that consist in addition to several protein components also ‘VariO:0493 ribosomal RNA’ in the subunits that are annotated as ‘VariO:0469 large subunit ribosomal RNA’ and ‘VariO:0470 small subunit ribosomal RNA’. m.1555A>G substitution in the small mitochondrial subunit 12S rRNA is associated with non-syndromic deafness [109]. The patients have also increased susceptibility to the ototoxic effects of aminoglycosides since several antibiotics target ribosomes and rRNAs.

‘VariO:0471 intron’ is a non-coding region located between exons in a pre-mRNA and are cleaved during maturation. Introns appear also in many non-protein coding RNAs from which they are cleaved during maturation. ‘VariO:0473 spliceosomal intron’ is cleaved at spliceosome. tRNA intron is cleaved by a tRNA splicing endonuclease, while ‘VariO:0472 self-splicing intron’ is removed autocatalytically. r.777_839del in *BTK* intron 3 position −1 causes exon skipping and is a spliceosomal intron variation [64]. ‘VariO:0403 Group I intron’ and ‘VariO:0404 group II intron’ are self-splicing introns that have catalytic activity to cleave and join the RNA chain. Group I introns appear in rRNA, mRNA and tRNA genes in bacterial genomes, in lower eukaryotes in mitochondrial and chloroplast genomes as well as in rRNAs. Group II introns can be found in all domains of life. ‘VariO:0468 transfer RNA’ molecules contain anticodon sequence that recognizes mRNA triplets. Loaded aminoacyl-tRNAs bring amino acids to ribosomes to be added to the elongated protein chain. Variants in these RNA molecules impair protein synthesis as an r.1616a>g substitution in tRNA-Val that causes MELAS syndrome [75].

Some 600 tRNA genes are coded by the human genome. Human mitochondrial genome codes for 22 tRNAs, the other tRNAs are imported nuclear-origin molecules. Several diseases are caused by variants in mitochondrial tRNA genes. PON-mt-tRNA is a predictor for disease relevance of mitochondrial tRNA variants [110]. Predictions for all the possible substitutions in all positions are available for all the 22 human mitochondrial tRNAs.

#### Location or origin of non-coding RNA

Certain RNA molecules are located to special compartments or cellular regions, there are also nuclear and mitochondrial RNAs, described by ‘VariO:0486 location or origin of non-coding RNA’. DM1-causing CTG repeats in the antisense RNA molecule [89] are in ‘VariO:0487 nuclear non-coding RNA’. MELAS syndrome-associated variations in tRNA-Val [75] are in ‘VariO:0488 mitochondrial non-coding RNA’. The expression of ‘VariO:0490 centromeric RNA’ is linked to chromosome segregation [111]. Some lncRNAs act at chromatin-modifying complexes as ‘VariO:0489 chromatin-related non-coding RNA’ to regulate gene expression [112]. ‘VariO:0501 RNA fragment’ describes RNA molecules that are products to RNA degradation. For example, tRNA-derived fragments are functional in some cellular responses and in cancers [113,114].

#### Size of non-coding RNA

‘VariO:0491 size of non-coding RNA’ can be classified into three categories. ‘VariO:0492 short non-coding RNA’ molecules are shorter than 200 nucleotides, often substantially shorter than the threshold. miR-140 which contains variants in skeletal dysplasia miRNA [77] is a short non-coding RNA. ‘VariO:0495 very long non-coding RNA’ chains are longer than 10 kb and ranging up to 1 Mbp. These molecules regulate expression on many genes, for a review see [115]. ‘VariO:0494 long non-coding RNA’ is a category for molecules between the two classes. Many methods have been developed to predict lncRNA interactions and disease-association. Studies for variation effects have started to emerge [116].

### Protein-coding RNA

There are several forms also of ‘VariO:0351 protein-coding RNA’. Alternative splicing is a common mechanism regulating gene expression [117] and increasing proteome complexity and can be detailed as ‘VariO:0411 alternatively spliced mRNA’ of ‘VariO:0331 alternative form of mRNA’. Exons are transcript regions that are part of mature mRNA. Exons occur in most eukaryotes. The number of exons varies greatly between genes. In humans, genes for histones contain just one single exon, they are not spliced at all. In the other end of the spectrum, *TTN* for titin contains 363 exons [118]. The shortest known human exon is just 2 bp, while the longest one is 27,303 bp long. The corresponding numbers for introns are 26 and 1,160,411 bp.

‘VariO:0460 pre-mRNA’ is matured to ‘VariO:0461 mRNA’ via several processing steps. pre-mRNAs constitute substantial part of heterogeneous nuclear RNA (hnRNA) and contain many very long RNA molecules. The longest human gene is for RNA-binding fox-1 homolog 1 *RBFOX1* of 2,473,592 bp, the longest mRNA is for *TTN*, 109,224 bp. Variations in ‘VariO:0352 coding region of mRNA’ lead to many types of protein variants, discussed in [4]. mRNA is formed by joining exons during splicing. Note that ‘VariO:0496 exon’ may contain non-coding RNA in the termini. These regions are annotated with ‘VariO:0353: non-coding region of mRNA’.

One of the mRNA maturation steps is excision of introns during splicing. Alternative splicing is common, some 95% of multiexon genes could undergo alternative splicing [119–121], but it is unclear how many forms are biologically relevant as many of them are extremely rare, restrained to a few cell types and may thus not be the explanation for the majority of complexity of proteome [122]. Combined RNA sequencing and proteomics data along with bioinformatic predictions indicated 72% of human genes to have alternative splice forms that could be translated to proteins [123]. Analysis of functionally distinct splice forms in over 700 human and mouse genes, biased towards literature notions of alternative splicing, indicated that just a small fraction of the transcripts was functionally distinct [124]. Depending on the criteria, 5% to 13% of human genes were shown to include such transcripts.

Although alternative splicing produces large numbers of variant proteins, alternative start and termination produce even wider range of variation [125]. They detected tissue-dependent transcripts for about half of the 18 000 investigated protein-coding genes and mainly due to alternative transcription start and termination. ‘VariO:0437 alternatively initiated mRNA’ of ALK receptor tyrosine kinase (*ALK*) is frequent in melanomas and appears also in some other cancer types [126]. The novel initiation codon appears in intron 19 and codes for three proteins of different sizes. Similarly, ‘VariO:0497 alternatively terminated mRNA’ affects produced protein product.

Addition of polyadenylation signals to 3ʹ end is one of mRNA maturation processes. Heterogeneity can appear at the polyadenylation tails and this can lead even to diseases as the poly-A tails are targets for miRNA regulation. ‘VariO:0356 alternatively polyadenylated mRNA’ due to single nucleotide variations can affect transcript length and gene expression [127]. Variations in ‘VariO:0352 coding region of mRNA’ lead to many types of protein variants, discussed in [4].

‘VariO:0462 circular RNA’ is a covalently closed single-stranded RNA ring. circRNAs are formed via splicing and can code for proteins. These molecules are formed when an upstream splicing acceptor joins with a downstream splice donor by back-splicing mechanism. circRNAs are common and have roles both in diseases and development [128]. hsa_circ_0124644 can be used as a biomarker for cardiovascular artery disease [129]. This circular RNA is thought also to be involved in disease pathogenesis.

Although DNA is the most common polynucleotide for genomic information in nature, ‘VariO:0456 RNA genome’ contains the genetic material in many viruses. ‘VariO:0457 RNA virus genome’ is usually single-stranded. According to the International Committee on Taxonomy of Viruses (ICTV) RNA viruses are classified to Group III, Group IV or Group V in the Baltimore classification system [130]. As RNA viruses are considered those which do not have a DNA intermediate during replication. Many common disease-causing viruses have RNA genome including SARS-CoV-2, influenza, hepatitis C, Ebola, and rabies viruses. Human immunodeficiency virus and some others have RNA genomes and replicative DNA intermediates and therefore are called retroviruses.

Viroids are the smallest known pathogens, they appear in plants. ‘VariO:0458 viroid genome’ contains just a single-stranded, circular RNA without any protein coating or other molecules. Viroids are classified as subviral agents by ICTV. *Coleus blumei* viroid 1 (CbVd-1) variants clone 1 (accession number MG767212) and clone B (DQ178395) differ at position 25 and have different seed-transmission frequencies, 30% to 0% [131]. Satellites are another group of subviral agents. They mainly affect plants. Their ‘VariO:0459 satellite genome’ contains genes for protein shell but they require helper virus to replicate. Variants leading to p.D35A and p.M98R substitution in the *Satellite panicum mosaic virus* coat protein Kansas isolate (SPMV-KS) affect interaction with the helper *Panicum mosaic virus* [132].

### VariO:0354 effect on posttranscriptional RNA modification

**Figure 4.**
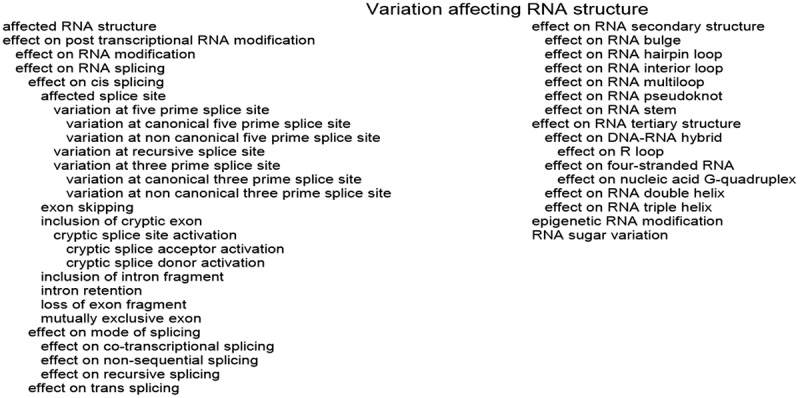
Terms describing structural variations. Note that details for affected RNA types are in Fig. 3. The hierarchy of the terms is indicated by indentation

Effect on post-transcriptional RNA modification includes variations that can be described either with ‘VariO:0498 effect on RNA modification’ or ‘VariO:0362 effect on RNA splicing’, see Fig. 4.

Both noncoding, including tRNAs, rRNAs, spliceosomal small RNAs, etc., and coding RNAs (mRNAs) frequently contain nucleotide modifications. Collectively the different RNA forms are known to have more than 100 distinct modifications, see MODOMICS database [38]. m.14692A>G in the mitochondrially encoded tRNA-Glu (GAA/G) gene *MT-TE* replaces highly conserved uridine r.55u>c in TΨC loop that is modified to pseudouridine and affects the conformation and stability of the tRNA molecule leading to maternally inherited diabetes and deafness [133]. The variant has ‘VariO:0354 effect on posttranscriptional RNA modification’ specifically on ‘VariO:0498 effect on RNA modification’.

**Figure 5.**
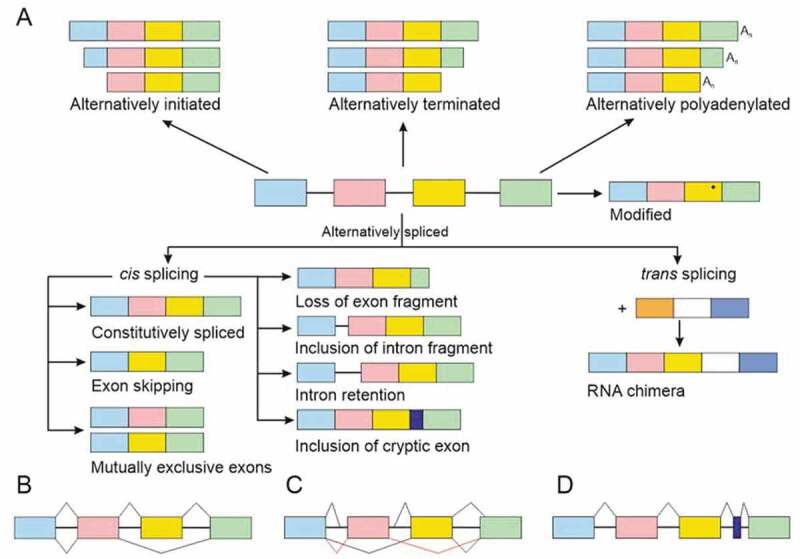
mRNA forms and mechanisms causing them. (A) The mRNA molecule (in the centre) can be modified in many ways. Exons are shown as boxes with different colours, introns are indicated with a thin line. mRNA molecules can have alternative initiation and termination positions, and the polyadenylation can start at different sites. mRNA bases can be modified. During splicing introns are cleaved. c*is*-Splicing is the most common splicing even and occurs within a single hnRNA molecule. In constitutive splicing all exons are included. Exon skipping means that one or more exons are excluded from the mature mRNA. It can appear also as mutually exclusive exons where only one of two exons is included to the final product. When a cryptic splice site is activated a new cryptic exon out of an intron may be included. Intron fragment or entire intron can be retained in the sequence. Variations can lead also to loss of exon fragment. In *trans*-splicing exons from different mRNA molecules are combined to form a chimeric RNA. (B) Constitutive splicing (top) and exon skipping (bottom). Exon skipping can occur due to several reasons. It may be normal variation between cells or tissues or dependent on the cellular developmental situation. Variations at splice site or at their surrounding, such as in exonic splicing enhancer, can lead to exon skipping. (C) Inclusion of intronic sequence to mature mRNA due to alternative 3ʹ acceptor (top left) or 5ʹ donor (top middle) splice sites, or because of novel splice site formation inside an intron (top right). The alternative splice sites can appear either on exon or intron. Mutually exclusive splicing (bottom) produces two forms that contain only one of two alternative exons (red and black lines). (D) Inclusion of cryptic exon due to variation at splice site or at a site activating the novel splice site

In addition to mRNAs, e.g., tRNAs, rRNAs, lncRNAs, ribozymes and circRNAs contain introns or spacers that are removed during maturation before ligating the ends of the chains. Variations at canonical and noncanonical splice sites and those introducing cryptic splice sites can alter mRNA structure and have ‘VariO:0362 effect on RNA splicing’. The ‘VariO:0509 effect on mode of splicing’ has three subterms that describe the type of splicing process affected (Fig. 5). Splicing of many transcripts occurs simultaneously with transcription [134] and variant can have a ‘VariO:0510 effect on co-transcriptional splicing’. Dystrophin (*DMD*) is the largest human gene, it contains 79 exons. Some of the introns are subject to non-sequential and recursive splicing [135], where variation can have ‘VariO:0512 effect on recursive splicing’. Recursive splicing means stepwise removal of an intron by several splicing events. Variation at intron eight donor site position +1 in collagen type I alpha 1 chain (*COL1A1*) causes osteogenesis imperfecta due to splicing defect [136]. The variation leads to production of five distinct splice forms, which are defective and have ‘VariO:0511 effect on non-sequential splicing’ by affecting the order at which introns are removed.

Fig. 5 depicts various mRNA splicing forms and mechanisms. A change that introduces a new splice site can cause ‘VariO:0505 inclusion of intron fragment’. c.801+2_801+3insT variation in *GLA* gene for galactosidase alpha leads to two aberrant transcripts [137]. In one, a novel donor splice site is created causing inclusion of 37 bp from intron to the mRNA. The patient has Fabry disease because complex glycosphingolipids are stored inside lysosomes resulting in a progressive multisystem disease. ‘VariO:0474 intron retention’ contains the entire intron sequence in the processed mRNA. A silent heterozygous substitution c.7464C>T in exon 44 of the von Willebrand factor (*VWF*) gene causes type 1 von Willebrand disease [138]. Intron 44 is retained in the mRNA. As the transcript contains premature stop codon, it is likely degraded and no protein is produced.

c.1029+384A>G transition to human serpin family G member 1 (*SERPING1*) gene creates a donor splice site in intron 6 and causes ‘VariO:0504 inclusion of cryptic exon’ leading to hereditary angioedema (HAE) type I [139]. This variant can be described more precisely with terms ‘VariO:0373 cryptic splice site activation’ and ‘VariO:0374 cryptic splice site donor activation’. This kind of inserted regions are typically transposed elements, most often Alu sequences. Formation of a new exon from intronic DNA sequence has been called in literature for exonization. If the variant had generated acceptor site, then ‘VariO:0375 cryptic splice acceptor activation’ would be used for annotation.

‘VariO:0513 mutually exclusive exon’ annotates situations where only one of two exons is included into mRNA. C>G substitution at position +19 in exon 10 of microtubule-associated protein tau *MAPT* gene affects splicing of mutually exclusive exons and causes frontotemporal dementia with Parkinsonism linked to chromosome 17 (FTDP-17) [140]. Mutually exclusive exons are also called for cassette exons. They are typically about the same size and are evolutionarily conserved [141].

Variants in ribosomal protein S6 kinase A3 (RPS6KA3) cause Coffin-Lowry syndrome (CLS) with variable phenotypes, e.g., with digital and facial anomalies as well as syndromic intellectual disability. c.613G>C in *RPS6KA3* causes partial exon skipping ‘VariO:0502 loss of exon fragment’ and leads to a premature termination codon [142]. The patient has also another transcript that codes for amino acid substitution in an important amino acid residue.

Substitution c.839+5G>A in *BTK* gene causes XLA due to ‘VariO:0502 exon skipping’ and deletion of 21 residues [64]. The deletion is in-frame and thus the protein sequence is retained after the deleted segment. This variant is classified also as a ‘VariO:0367 variation at five prime splice site’ of type ‘VariO:0369 variation at non canonical five prime splice site’. c.392–2A>C in *BTK* causing r.392_520del [65] is a ‘VariO:0370 variation at three prime splice site’ of type ‘VariO:0372 variation at canonical three prime splice site’. Introns can be very large, the longest in human is over 1 million bp. Large introns can contain cryptic recursive splice sites which facilitate stepwise removal of introns [143]. Alteration to such site is a ‘VariO:0366 variation at recursive splice site’.

All the examples above are of type ‘VariO:0365 effect on cis splicing’. ‘VariO:0376 effect on trans splicing’ is used to describe splicing and ligation of two mRNA molecules producing a chimeric molecule. Trans splicing is actively investigated as a gene therapy modality to correct errors in mRNAs by generating RNA chimeras [144].

Lots of computer predictors have been released for various aspects of splicing. Tools for the effects of variants on splicing, including acceptor and donor splice sites, exonic sequences, exonic and intronic splicing silencers and enhancers, branch point sequences and polypyrimidine tracts were reviewed in [145]. There are several tools for 5ʹ splice sites, but very limited amount for branch point sequences [145]. Predictors for splice site identification have been around for two decades. Predictions for all possible single nucleotide substitutions in positions −3 to +8 at 5ʹ splice site and in positions −12 to +2 at 3ʹ slice site are available from dbscSNV [146].

Several methods have been developed for RNA modification site [147], usually specific tools for each type of modifications, including *N*^6^-methyladenosine (m^6^A) [148], 5-methylcytosine (m^5^C) [149], pseudouridine [150,151] and others. Performance of methods for *N*^1^-methyladenosine (m^1^A) and m^6^A modifications have been benchmarked [152]. tRNAmodpred is an example of an RNA type-specific modification predictor [153].

### VariO:0382 effect on RNA secondary structure

**Figure 6.**
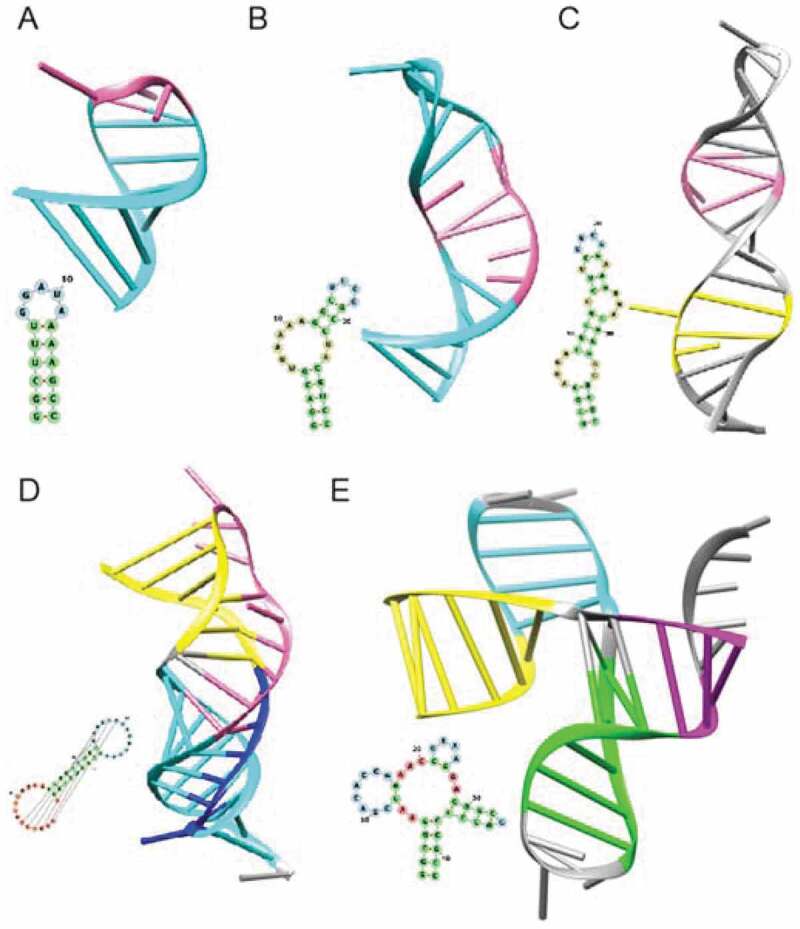
Three dimensional and simplified ladder models for three-dimensional structures of RNA secondary structural elements. (A) Stem (cyan) and loop (pink) connecting the strands in the loop of 3ʹ conserved region of eel LINE element UnaL2 (PDB entry 1wks [154]). (B) Bulge (pink) in non-coding prohead RNA from GA1 bacteriophage, which is involved in metal ion binding (2nci [155]). (C) Asymmetric internal loops A (yellow) and B (pink) in SL1 domain in human immunodeficiency virus HIV1 packaging signal (1m5l [156]). HIV is an RNA virus. (D) Pseudoknot in human telomerase RNA (2k96 [157]. The two stems are indicated in yellow and cyan, and the two loops in pink and dark blue, respectively. (E) Multiloop structure in RNA tertiary domain essential to hepatitis C virus (HCV) internal ribosome entry site (IRES) -mediated translation initiation (1kh6 [158]). The four stems are indicated in cyan, red, green and yellow. In the case of ensemble of structures, the representative chain was selected. The 2D structures were drawn with forna based on force-directed graph layout [159] and 3D structures were drawn with UCSF Chimera [160]

The primary RNA structure (sequence) forms secondary structural elements that are central components of the three-dimensional tertiary structure. The folded chain can then form quaternary structures together with RNA and other molecules. There are six types of RNA secondary structures (Fig. 4). Stem is formed by complementary bases binding together into a double-stranded structure (Fig. 6A). The stabilizing hydrogen bonds in the stems are similar to those in double-stranded DNA. Hairpin loops appear between stem-forming regions and do not contain stabilizing interactions (Fig. 6A). In a bulge one of the strands in a stem region contains a base or bases that do not form a pair with the other strand (Fig. 6B). Internal loop contains mismatching bases in both strands (Fig. 6C). The mismatches can of the same or different lengths. Pseudoknot is a special structure where three strands (parts of the same chain) come together (Fig. 6D). Multiloop is the most complicated of the secondary structures. Two or more double-stranded stems meet in a multiloop (Fig. 6E).

Variations in the secondary structural elements have various effects. C>G substitution in *MAPT* gene exon modifies the structure of stem in the mRNA and increases its stability [140]. FTDP-17 is caused by changing the ratio of alternative proteoforms containing either three or four microtubule-binding repeat domains due to ‘VariO:0137 effect on RNA tertiary structure’ of ‘VariO:0386 effect on RNA stem’ [140].

Some variations within hairpin loops cause conformational alterations [161] and have ‘VariO:0387 effect on RNA hairpin loop’. RNA bulges show a linear correlation between the size of the bulged loop and its stability [162] and have ‘VariO:0384 effect on RNA bulge’. Yeast *Saccharomyces cerevisiae* ribosomal protein L30 represses its own splicing and translation. Single and multiple variations affect both protein affinity and repression [163] having ‘VariO:0385 effect on RNA interior loop’. Asymmetric loop positions show differential tolerance for substitutions. Positions 55 and 57 in *L30* transcript tolerate alterations, while changes at sites 10, 11, 12, 58 or 59 have marked effect on binding and regulation [163].

Telomerase complex maintains chromosome telomere length and stability. Telomerase RNA component has a highly conserved pseudoknot, variations in which disrupt the structure and abolish telomerase activity due to ‘VariO:0500 effect on RNA pseudoknot’ [164]. Even ‘VariO:0383 effect on RNA multiloop’ can be induced by variants.

### VariO:0137 effect on RNA tertiary structure

**Figure 7.**
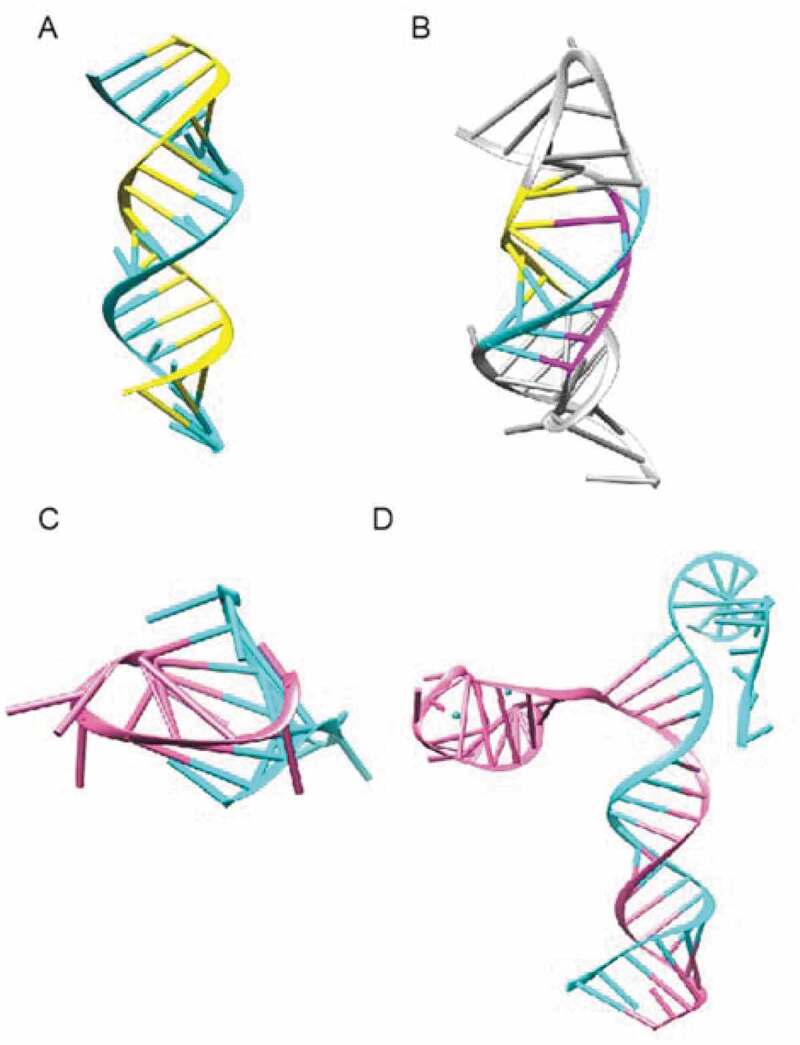
RNA structures. (A) Double-stranded RNA helix (6IA2 [167]) in a self-complementary RNA duplex recognized by bacteriophage Mu zinc finger protein Com. (B) RNA triple helix in telomerase TER ribonucleoprotein complex RNA component (2K95 [157]). (C) G-quadruplex is a form of four-stranded RNA. The structure is for human telomeric RNA (2KBP [166]). (D) RNA-DNA complex of Cpf1 endonuclease R-loop complex (5MGA [170]). RNA chain in pink and DNA chains in cyan. The large protein component of the complex is not shown

RNA molecules fold into three-dimensional and quaternary structures and form a large number of structural forms (Fig. 4). RNA double helix is formed when different parts of a RNA molecule hybridize and fold together or when two chains bind complementarily. C>G substitution in exon 10 of *MAPT* gene [140] is an example of ‘VariO:0381 effect on RNA double helix’ (Fig. 7A). Short triple-helical RNA stretches have been found from a number of proteins [165]. These regions are structurally and functionally important, e.g., in telomerase TER RNA component (Fig. 7B) where variants have ‘VariO:0425 effect on RNA triple helix’. Similar to DNA, RNA can form also four-stranded structures, such as G-quadruplex [166] (Fig. 7C). Changes to these have ‘VariO:0426 effect on four-stranded RNA’ of type ‘VariO:0173 effect on nucleic acid G-quadruplex’.

DNA and RNA chains can bind complementarily and form hybrids. R loop is formed during transcription, it consists of a DNA:RNA hybrid and a displaced single-stranded DNA (Fig. 7D). These loops are unstable and targets for nuclease cleavage [168]. They are linked to human diseases, including trinucleotide repeat-associated diseases [169]. Changes to these hybrids can have a ‘VariO:0431 effect on R loop’ [170] which is a form of ‘VariO:0424 effect on DNA-RNA hybrid’. R-loop DB [171] is a resource for both predicted and detected R loops in 8 organisms, including humans.

RNA sugar component is modified in a number of instances. Position 34 in the anticodon wobble position is modified in mammalian tRNAs. The modifications include queuosine in tRNA-Asn and tRNA-His, mannosyl-queuosine in tRNA-Asp, and galactosyl-queuosine in tRNA-Tyr [172]. These kinds of variations are annotated to have ‘VariO:0361 RNA sugar variation’.

‘VariO:0438 epigenetic RNA modification’ has been included to describe potential epigenetic RNA changes. Epigenetic changes are heritable traits that do not change the DNA sequence. Inherited epigenetic changes are known in DNA and protein. In RNA field epigenetics is used in a misleading and non-systematic way, i.e., for RNA modifications (‘epitranscriptomics’). These changes are not inherited and are thus not epigenetic. Gene expression regulation, e.g., by non-coding RNAs is not an epigenetic trait either, it is one form of regulation. These changes are annotated with ‘VariO:0354 effect on post-transcriptional RNA modification’ and ‘VariO:0498 effect on RNA modification’, similar to post-translational modifications in proteins. There are a few verified examples where short RNA molecules are involved in epigenetics, however even these are not ‘VariO:0438 epigenetic RNA modification’, as the epigenetic effect is not on RNA level.

## Conclusion

RNA related research is advancing at long paces. New RNA forms are reported frequently and novel insights are obtained on the function and importance of the various transcripts. Thus, there is immediate need for systematic description of RNA related information to facilitate data mining, integration and analysis also from several sources. Comprehensive conceptualization of RNA variations was implemented into VariO to facilitate detailed description of all kinds of RNA variants, effects, consequences and mechanisms. Consistent annotations can be made with VariOtator tool [6]. RNA terms can be used together with terms from other systematics to enrich the information content. VariO terms at several levels can be combined for this purpose.
